# Editorial: Obesity and gastrointestinal cancer

**DOI:** 10.3389/fsurg.2022.1013611

**Published:** 2022-10-10

**Authors:** Irene Lidoriki, Efstathia Liatsou, Dimitrios Schizas, Antonios Athanasiou, Konstantinos G. Toutouzas, Maximos Frountzas

**Affiliations:** ^1^ Department of Nutrition and Dietetics, Harokopio University, Athens, Greece; ^2^ Department of Clinical Therapeutics, Alexandra General Hospital, National and Kapodistrian University of Athens, School of Medicine, Athens, Greece; ^3^ First Department of Surgery, Laikon General Hospital, National and Kapodistrian University of Athens, School of Medicine, Athens, Greece; ^4^ Department of Surgery, Queen Elizabeth Hospital Birmingham, Birmingham, United Kingdom; ^5^ First Propaedeutic Department of Surgery, Hippocration General Hospital, National and Kapodistrian University of Athens, School of Medicine, Athens, Greece

**Keywords:** gastrointestinal, cancer, obesity, fat, adipose

**Editorial on the Research Topic** Obesity and gastrointestinal cancer By Lidoriki I, Liatsou E, Schizas D, Athanasiou A, Toutouzas KG, Frountzas M. (2022) Front. Surg. 9: 1013611. doi: 10.3389/fsurg.2022.1013611

Obesity is a worldwide “epidemic” which has been associated to various diseases, such as cardiovascular dysfunction and cancer (1). Excess adiposity has been involved in the pathophysiology of several types of gastrointestinal (GI) cancer, like esophageal, gastric, colorectal cancer and hepatobiliary cancer. This association could be likely attributed to obesity-associated chronic inflammation, adipokines secreted from adipose tissue and altered metabolism of sex hormones (2). It is worth mentioning that a molecular cross-talk between human adipocytes and innate immunity cells has been proved, and their alteration in obesity and colorectal cancer might lead to immune dysfunctions, thus setting the basis for cancer development (3). Excessive adipose tissue has been associated to high levels of triglycerides (TGs) and low-density lipoproteins (LDL), as well as remarkable tissue resistance to insulin. Consequently, hyperinsulinemia is caused, which seems to play a crucial role in oncogenesis (4).

Many studies have highlighted the relationship between high body mass index (BMI) and cancer's prevalence and prognosis (5–7). However, other studies have shown that cancer prognosis is negatively affected by low body weight. For instance, according to Song et al. (2022), nutritional risk index (NRI), which was based on ideal body weight, present body weight and serum albumin, was identified as a potential prognostic factor for patients with stage III gastric cancer. A possible explanation for this discrepancy could be that body weight and BMI are not the only indicative obesity markers associated to increased cancer risk and impaired survival. Other measures of excessive adiposity, such as body composition, especially the amount of white adipose tissue, are more consistently correlated to tumorigenesis. Visceral Adipose Tissue (VAT) which is accumulated around abdominal organs is more strongly associated with insulin resistance, compared to Subcutaneous Adipose Tissue (SAT). As a result, a high VAT/SAT index is linked to insulin resistance, dyslipidemia and development of metabolic syndrome.

High insulin levels lead to increased levels of Insulin-like Growth Factor-1 (IGF-1), which activates signaling pathways for cell proliferation, cell differentiation, cell growth and apoptosis. Recent data have shown that decreased levels of circulating insulin and IGF-1 are associated with reduced risk for colon cancer (8). Moreover, increased VAT deposition is associated to secretion of interleukin 6 (IL-6) at higher concentrations in comparison with SAT. VAT is responsible for the synthesis and secretion of several hormones directly to the portal circulation, causing a direct effect on liver's function.

On the other hand, endocrine products from SAT, which mainly involve leptin and adiponectin in higher concentrations than VAT inside peritoneal cavity, are released in the systemic circulation (9). Apart from cancer predisposition, adipose tissue might also affect postoperative outcomes. Wen-Bin Wang et al. (2021) investigated the impact of body composition and physical function on quality of life (QoL) after gastrectomy for gastric cancer. One month after surgery, patients with a higher subcutaneous fat area (SFA) at diagnosis presented more deteriorated QoL. In addition, higher visceral fat area (VFA) content was associated to a decline in cognitive and role functioning, whereas it was correlated to greater anxiety at the same time point. Therefore, these patients might benefit from a personalized plan to reduce SFA and improve physical function. However, postoperative outcomes, as well as QoL, might be affected by the operative technique used in gastrectomy; thus, the ideal technique should be identified, according to Weidong Wang et al. (2022).

Obese patients have increased levels of circulating leptin, that is secreted by white adipocytes, and present dysregulated production of leptin receptors (LRs), which might play a role in the development of several malignancies. The role of LRs in carcinogenesis is further supported by the association between high level of LRs in the tumor cell tumor and tumor clinical stage and prognosis, especially in esophagus, stomach, colon and hepatobiliary system (10–12). Pappas-Gogos et al. (2022) investigated the association between vascular endothelial growth factor (VEGF) and leptin in patients with intestinal metaplasia of the stomach and metabolic syndrome. Patients with intestinal metaplasia and metabolic syndrome had elevated levels of VEGF, while leptin levels were predominantly associated to metabolic syndrome, that has been associated to precancerous lesions of GI tract, such as Barrett esophagus.

Moreover, according to the review of Liu et al. (2022), there is emerging evidence that increased fecal bile acids, as a result of excessive body weight and/or high intake of fatty acids, have been implicated in the pathogenesis of colorectal cancer. Different mechanisms for this association include proliferation and apoptosis of colonic epithelial cells, increased intestinal permeability and alterations of the intestinal microbiome. Bile acids stimulate the growth of bile acid-metabolizing bacteria, whereas they are associated to decreased proliferation of bile acid-sensitive bacteria (13, 14).

Accumulated experimental data have shown that hypoxia of adipocytes makes them prone to chronic inflammation, as suggested by the presence of crown-like macrophages, accelerated adipocyte death caused by dysregulated mitochondria and impaired response of endoplasmic reticulum due to high stress levels (15). Hypoxia plays a significant role in cancer development and has been correlated to poor prognosis. Yang et al. (2022) investigated the role of five hypoxia-related genes in rectal cancer and the effect of hypoxia on neutrophil-mediated immunosuppression. Overall survival of the high-risk group was significantly higher compared to the low-risk group. In addition, high-risk group was associated to lower tumor purity, higher immune and stromal score, higher neutrophil count, and lower activated memory CD4+ T-cells. Anillin actin-binding protein (ANLN), which inhibits cancer, and SRPX (Sushi Repeat Containing Protein X-Linked), which promotes cancer, might be potential therapeutic targets for rectal cancer.

Despite all existing evidence related to this emerging topic, there are still many aspects to be elucidated, especially the impact of other proteins expressed in white adipose tissue, such as chemerin and adipocyte fatty acid-binding protein 4 (FABP4), which have been correlated to poor cancer prognosis. Different types of malignancy could lead to several lethal complications, such as ileus perforation and peritonitis due to advanced-stage Non-Hodgkin lymphoma (NHL), as Yong et al. (2022) described. Therefore, future research should be designed towards identifying new therapeutic targets and achieving prompt treatment of patients with early diagnosed tumors.
